# Oral and Intravenous Iron Therapy Differentially Alter the On- and Off-Tumor Microbiota in Anemic Colorectal Cancer Patients

**DOI:** 10.3390/cancers13061341

**Published:** 2021-03-16

**Authors:** Oliver Phipps, Hafid O. Al-Hassi, Mohammed N. Quraishi, Edward A. Dickson, Jonathan Segal, Helen Steed, Aditi Kumar, Austin G. Acheson, Andrew D. Beggs, Matthew J. Brookes

**Affiliations:** 1 Research Institute in Healthcare Science, Faculty of Science and Engineering, University of Wolverhampton, Wolverhampton WV1 1LY, UK; h.omar6@wlv.ac.uk (H.O.A.-H.); helen.steed@nhs.net (H.S.); aditikumar@nhs.net (A.K.); matthew.brookes@nhs.net (M.J.B.); 2 Surgical Research Laboratory, Institute of Cancer & Genomic Science, University of Birmingham, Birmingham B15 2TQ, UK; M.N.Quraishi@bham.ac.uk (M.N.Q.); a.beggs@bham.ac.uk (A.D.B.); 3 Microbiome Treatment Centre, University of Birmingham, Birmingham B15 2TT, UK; 4 Department of Gastroenterology, University Hospitals Birmingham NHS Foundation Trust, Birmingham B15 2TH, UK; 5 NIHR Biomedical Research Centre in Gastrointestinal and Liver Diseases, Nottingham University Hospitals NHS Trust, Nottingham NG7 2UH, UK; edward.dickson1@nottingham.ac.uk (E.A.D.); austin.acheson@nottingham.ac.uk (A.G.A.); 6 Department of Colorectal Surgery, Nottingham University Hospitals NHS Trust, Nottingham NG5 1PB, UK; 7 Department of Gastroenterology, St. Mark’s Hospital, Harrow HA1 3UJ, UK; jonathansegal1@nhs.net; 8 Department of Gastroenterology, Royal Wolverhampton Hospitals NHS Trust, Wolverhampton WV10 0QP, UK

**Keywords:** iron supplementation, iron deficiency, anemia, gut microbiome, 16S rRNA, tumor microbiota, colorectal cancer

## Abstract

**Simple Summary:**

Anemia is commonly associated with colorectal cancer and often requires intervention with therapeutic iron. However, iron is required for growth by the majority of colonic bacteria, leading to competition for free luminal iron. Hence, this leaves the potential for the route of iron administration to lead to differential gut bacterial populations. This study aimed to investigate the differences in on- and off-tumor bacterial populations following oral and intravenous therapy in anemic colorectal cancer patients. The following iron therapies were shown to be differential to bacterial diversity, microbial populations, and predictive metagenomics, inferring that oral iron-treated patients may have a potentially more procarcinogenic microbiota compared to intravenous iron-treated patients. Overall, this suggests that intravenous iron may be a more beneficial treatment for anemia in colorectal cancer, in order to limit microbial perturbations associated with oral iron.

**Abstract:**

Iron deficiency anemia is a common complication of colorectal cancer and may require iron therapy. Oral iron can increase the iron available to gut bacteria and may alter the colonic microbiota. We performed an intervention study to compare oral and intravenous iron therapy on the colonic tumor-associated (on-tumor) and paired non-tumor-associated adjacent (off-tumor) microbiota. Anemic patients with colorectal adenocarcinoma received either oral ferrous sulphate (*n* = 16) or intravenous ferric carboxymaltose (*n* = 24). On- and off-tumor biopsies were obtained post-surgery and microbial profiling was performed using 16S ribosomal RNA analysis. Off-tumor α- and β-diversity were significantly different between iron treatment groups. No differences in on-tumor diversity were observed. Off-tumor microbiota of oral iron-treated patients showed higher abundances of the orders Clostridiales, Cytophagales, and Anaeroplasmatales compared to intravenous iron-treated patients. The on-tumor microbiota was enriched with the orders Lactobacillales and Alteromonadales in the oral and intravenous iron groups, respectively. The on- and off-tumor microbiota associated with intravenous iron-treated patients infers increased abundances of enzymes involved in iron sequestration and anti-inflammatory/oncogenic metabolite production, compared to oral iron-treated patients. Collectively, this suggests that intravenous iron may be a more appropriate therapy to limit adverse microbial outcomes compared to oral iron.

## 1. Introduction

Perturbations in gut bacterial populations are characteristic of colorectal cancer, with the presence of pathogenic indigenous bacterial species at the expense of protective probiotic species [1]. Driver bacteria are involved in the initiation of colorectal cancer. These driver bacteria are gradually outcompeted by opportunistic passenger bacteria, which have a selective advantage within the newly defined tumor-microenvironment. These passenger bacteria can potentially either promote or hinder tumor progression, depending on whether pathogenic or probiotic bacterial populations flourish [2,3]. Alongside this, the gut microbiota has been shown to be altered in response to many commonly used orally administered non-antibiotic drugs [4]. Hence, this allows the potential for the route of iron administration in anemic colorectal cancer patients to lead to differential gut microbial populations. Therefore, depending upon which bacterial populations thrive, the route of iron administration may support colorectal cancer progression [2,5].

Iron is essential for the growth and development of the vast majority of gut bacteria, with pathogenic bacteria tending to have heightened iron acquisition mechanisms [6]. Hence, in anemic colorectal cancer patients, the use of oral iron supplementation has the potential to alter bacterial populations of the colorectal tumor-associated (on-tumor) microbiota, as well as the non-tumor-associated mucosal (off-tumor) microbiota, through increasing gut luminal iron concentration [7,8]. This may suggest that a parenteral route of iron administration may be more beneficial to treat anemia, without increasing iron availability to colonic bacteria. Initial data from the intravenous iron in colorectal cancer associated anemia (IVICA) trial suggest that intravenous iron may be more beneficial than oral iron in treating pre-operative anemia [9]. However, results from the preoperative intravenous iron to treat anemia before major abdominal surgery (PREVENTT) trial has put doubt over the efficacy of intravenous iron pre-operatively [10]. Hence, the most beneficial treatment of anemia pre-operatively, oral or intravenous iron, remains uncertain. This study intended to contribute to the discussion over the route of iron administration in anemic colorectal cancer patients through assessing the gut microbial outcomes following each iron therapy. We hypothesized that oral iron would promote a more procarcinogenic gut microbiota, relative to intravenous iron, potentially through increasing gut luminal iron concentration.

Current research presents conflicting evidence concerning the effect of iron supplementation in murine studies of gut bacterial diversity [11,12]. However, to our knowledge, a comparison of gut bacterial populations following oral and intravenous iron therapy in human studies of colorectal cancer has yet to be explored. This pilot intervention study aimed to compare the outcomes of oral and intravenous iron therapy on the on- and off-tumor microbiota, in order to assess which therapy is more beneficial to treat anemia in iron-deficient colorectal cancer patients.

## 2. Materials and Methods

### 2.1. Study Population

From the IVICA trial, a total of 40 anemic patients with non-metastatic colorectal adenocarcinoma were randomized to receive either oral ferrous sulphate 200 mg twice a day (*n* = 16) or intravenous iron (ferric carboxymaltose—Ferinject™; Vifor Pharma, Glattbrugg, Switzerland) dosed by weight and hemoglobin in accordance with the summary of product characteristics (*n* = 24) [9]. Treatment was administered at least 2 weeks pre-operatively and anemia was defined as having a hemoglobin level 10 g/L below the sex-specific World Health Organization definition (women ≤ 120 g/L, men ≤ 130 g/L). The duration of iron treatment and inclusion hemoglobin for treatment groups are presented in the patient demographics in Table 1. To limit the inclusion of non-iron-deficient anemic individuals, those with pre-existing hematological disease, renal failure, or undergoing current chemotherapy were not eligible for the trial. Colorectal tumor biopsies and paired tumor-adjacent colonic mucosal tissue biopsies were obtained post-surgery.

### 2.2. DNA Extraction and 16S rRNA Amplicon Sequencing

Microbial DNA was extracted from colorectal tumor biopsies and paired tumor-adjacent mucosal colonic tissue biopsies using a modified protocol of Qiagen All Prep DNA/RNA Mini Kit (Qiagen, Hilden, Germany). Biopsies were mechanically lysed using 5-mm steel beads (Qiagen) and 0.1-mm Zirconia/Silica beads (Strateck, Suffolk, UK) with a TissueLyser (Qiagen), followed by enzymatic and heat lysis. Extracted microbial DNA was used for 16S ribosomal RNA (rRNA) gene amplification and sequencing to determine the mucosal-adherent microbiota according to the Earth Microbiome project protocol [13]. Using primers targeted to the V4 region (515F-806R), the 16S rRNA genes were amplified in technical triplicates. This was performed using a single-step, single-indexed polymerase chain reaction (PCR). DNA extraction and 16S rRNA gene PCR were both performed in batch with appropriate multiple reagent-based negative controls. Paired-end sequencing (2 × 250 base pairs) was completed in a single batch using the Illumina MiSeq platform (Illumina, San Diego, CA, USA).

### 2.3. Statistical Analysis

Microbial analysis was performed using the Quantitative Insight into Microbial Ecology 2 (QIIME2) pipeline [14]. Bacterial taxonomy was assigned using the Silva-132-99% operational taxonomic units (OTUs) database [15]. Intra-sample comparisons (α-diversity) were assessed using the Mann–Whitney U test comparing variation in the abundance-based coverage estimator (ACE), Chao1, Shannon diversity index, Faith’s phylogenetic diversity, and observed OTUs. Inter-sample comparisons (β-diversity) were assessed using permutational multivariate analysis of variance (PERMANOVA) comparing Bray–Curtis dissimilarity and Jaccard similarity using principal coordinate analysis (PCoA). α-diversity indices and PCoA were plotted using the R package “ggplot2” [16]. Comparison of relative abundances of taxa between treatment groups and sample types was performed using a linear discriminant analysis (LDA) effect size (LEfSe); taxa with an LDA score greater than 2 with a *p*-value ≤ 0.05 were considered statistically significant [17]. Predictive functional profiles from microbial populations were derived using Phylogenetic Investigation of Communities by Reconstruction of Unobserved States 2 (PICRUSt2), using enzyme classification pathway analysis. Differences in predictive enzyme abundances were assessed using Statistical Analysis of Metagenomic Profiles (STAMP). Corrected q-values were calculated following multiple testing correction using the Storey false discovery rate. q-values ≤ 0.05 between treatment groups were considered statistically significant [18,19,20].

## 3. Results

### 3.1. On- and Off-Tumor Bacterial Diversity Following Iron Therapy

**Figure 1:**
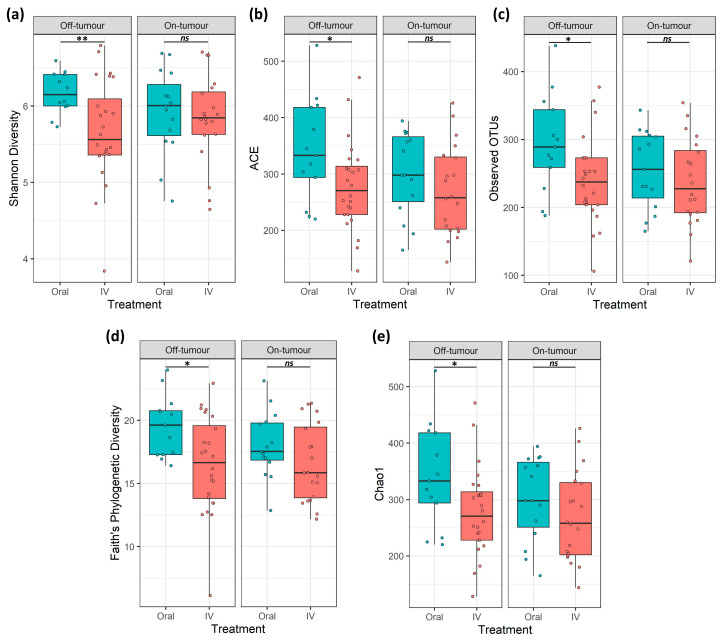
On- and off-tumor α-diversity is differentially altered following iron therapy. α-diversity metrics (**a**) Shannon diversity, (**b**) abundance-based coverage estimate (ACE), (**c**) observed operational taxonomic units (OTUs), (**d**) Faith’s phylogenetic diversity, and (**e**) Chao1. Off-tumor diversity metrics were significantly greater in oral iron compared to intravenous (IV) treated patients. On-tumor diversity showed no significant differences between oral and IV iron-treated patients (* *p* ≤ 0.05, ** *p* ≤ 0.01, ns *p* > 0.05).

**Figure 2:**
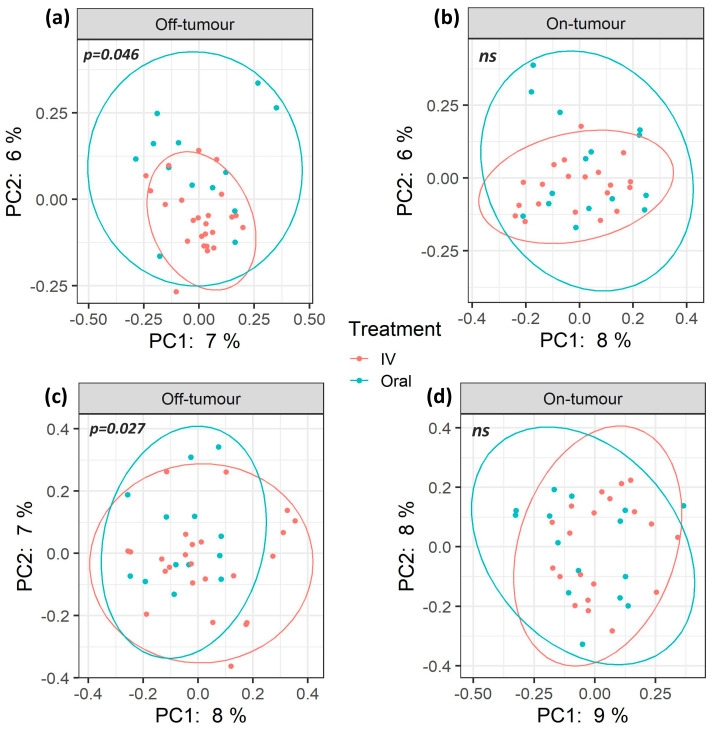
β-diversity of oral and intravenous iron-treated patients differs between on- and off-tumor microbiota. Principle coordinate analysis (PCoA) plots based on Jaccard (**a**,**b**) and Bray–Curtis (**c**,**d**) distances. Plot ellipses represent 95% confidence incidence for group clusters. The off-tumor β-diversity shows significantly distinct bacterial community clusters between oral and intravenous (IV) iron treatments (**a**; *p* = 0.046, **c**; *p* = 0.027. On-tumour β-diversity shows no significant differences (*ns*) between iron treatment (**b**,**d**).

A total of 7.9 million reads (an average of 109,777 reads/sample) and 2367 features were obtained following quality control, and a subsampling depth of 8000 reads/sample was chosen following rarefaction. Comparison of α-diversity metrics shows that patients treated with oral iron have a significantly higher bacterial diversity (Shannon diversity and Faith’s phylogenetic diversity), richness (observed OTUs), and abundance (Chao1 and ACE) within their off-tumor microbiota, relative to those treated with intravenous iron therapy (Figure 1; *p* < 0.05). Consistent with this, Jaccard similarity and Bray–Curtis dissimilarity assessed β-diversity between treatment groups and showed that within the off-tumor microbiota, oral and intravenous iron formed significantly different bacterial community clusters (Figure 2; *p* < 0.05).

The on-tumor microbiota of these patients showed no differences in α-diversity between iron treatment groups (Figure 1; *ns*). Along with this, no significant differences in β-diversity were observed (Figure 2: *ns*). Taken together, this suggests that oral iron and intravenous iron-treated patients show significantly different bacterial diversity in their tumor-adjacent microbiota. However, the tumor-associated microbiota showed a consistent bacterial diversity between iron treatments.

### 3.2. Oral and Intravenous Iron-Treated Patients Show Differing Bacterial Communities

**Figure 3:**
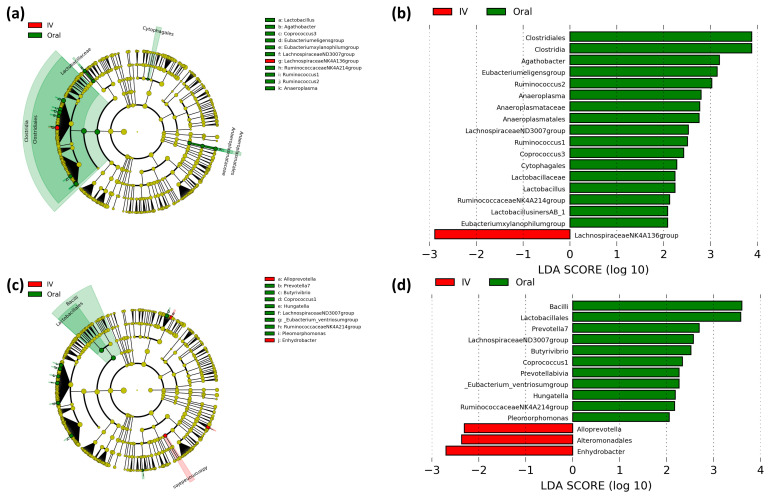
Linear discriminant analysis (LDA) effect size (LEfSe) comparing iron therapy on bacterial taxa in the on- and off-tumor microbiota. LEfSe cladogram and histogram of LDA scores for differentially abundant bacterial taxa between oral and intravenous (IV) iron-treated patients in off-tumor (**a**,**b**) and on-tumor (**c**,**d**) microbiota. Differentially abundant taxa from phylum to genus taxonomic levels were included. Taxa and nodes highlighted in green were more abundant in oral and red in IV iron-treated patients. Taxa with an LDA > 2 with a *p*-value ≤ 0.05 were considered statistically significant.

Differences in microbial populations between oral and intravenous iron-treated patients were assessed using LEfSe to determine bacterial taxa that are significantly enriched between treatments (Figure 3). The off-tumor microbiota of oral iron-treated patients showed a greater abundance of the Clostridia class, Clostridiales, Anaeroplasmatales, and Cytophagales orders, *Lactobacillaceae* and *Anaeroplasmataceae* families, and *Lactobacillus*, *Agathobacter*, *Coprococcus 3*, *Eubacterium eligens* group, *Eubacterium xylanophilum* group, *Lachnospiraceae ND3007* group, *Ruminococcaceae NK4A214* group, *Ruminococcus 1*, *Ruminococcus 2*, and *Anaeroplasma* genera, whereas in the intravenous iron group there was a higher abundance of the *Lachnospiraceae NK4A136* group genus (Figure 3a,b).

The on-tumor microbiota in oral iron-treated patients showed a greater abundance of the Bacilli class, Lactobacillales order, and *Prevotella 7, Butyrivibrio, Coprococcus 1, Hungatella*, *Lachnospiraceae ND3007* group, *Eubacterium ventriosum* group, *Ruminococcaceae NK4A214* group, and *Pleomorphomonas* genera, whereas the on-tumor microbiota of intravenous iron-treated patients showed a higher abundance of the Alteromonadales order and *Alloprevotella* and *Enhydrobacter* genera (Figure 3c,d). These results suggest that the on- and off-tumor microbiota show differential responses to iron therapy with oral iron leading to a more prominent change in bacterial taxa in the tumor-adjacent compared to the tumor-associated microbiota.

### 3.3. Differential Predictive Enzymatic Pathways between Iron Treatment Groups

**Figure 4:**
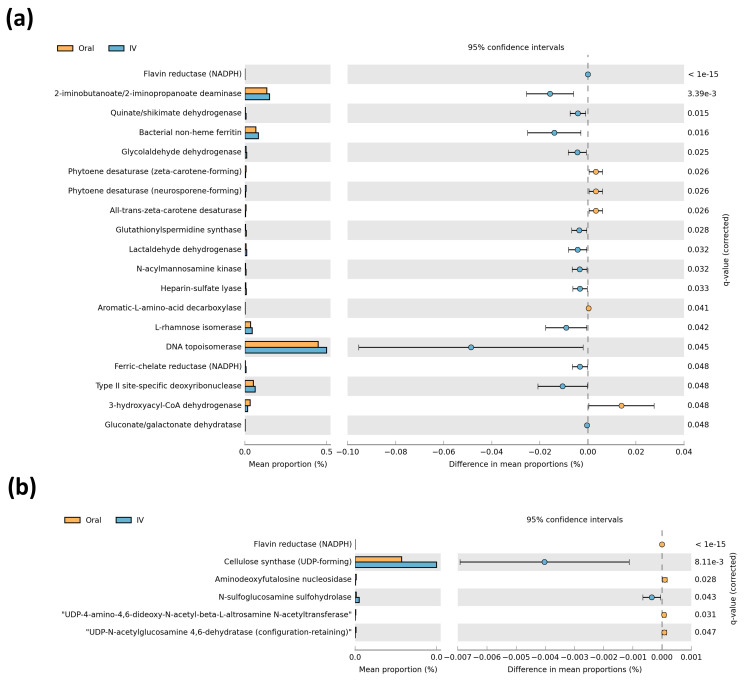
Comparison of predictive metagenomics between iron treatment groups. Predictive metagenomic analysis of enzyme classification abundances between oral and intravenous (IV) iron treatments, based on off-tumor (**a**) and on-tumour (**b**) microbial populations. Corrected q-values were calculated following multiple testing correction using Storey false discovery rate. q-values ≤ 0.05 were considered statistically significant.

In order to infer the metagenomic pathways associated with microbial profiles, we performed a PICRUSt2 metagenomic analysis using predicted enzyme classification abundances based on 16S rRNA bacterial populations. These showed a large difference in predicted microbial enzymes between iron treatments in the off-tumor microbiota (Figure 4a), while fewer differences in the on-tumor microbiota were observed (Figure 4b). The off-tumor microbiota were associated with increased abundance of iron-related enzymes, bacterial non-heme ferritin, and ferric-chelate reductase, in intravenous iron-treated patients compared to oral iron. Along with this, the off-tumor microbiota of intravenous iron-treated patients showed an increased abundance of enzymes involved in the production of anti-inflammatory metabolites, including lactaldehyde dehydrogenase and 2-iminobutanoate/2-iminopropanoate deaminase (Figure 4a).

The on-tumor microbiota was associated with an increased abundance of enzymes involved in anti-inflammatory and colorectal cancer-protective metabolite production, including cellulose synthase and N-sulfoglucosamine sulfohydrolase, in intravenous iron-treated patients (Figure 4b). Overall, the predictive metagenomic results suggested that the on- and off-tumor microbiota profiles associated with intravenous iron-treated patients are involved in regulating iron metabolism and producing metabolites involved in inhibiting intestinal inflammatory and colorectal cancer, compared to oral iron-treated patients.

### 3.4. Paired On- and Off-Tumor Microbiota Show Varying Microbial Communities Following Oral and Intravenous Iron Therapy

**Figure 5:**
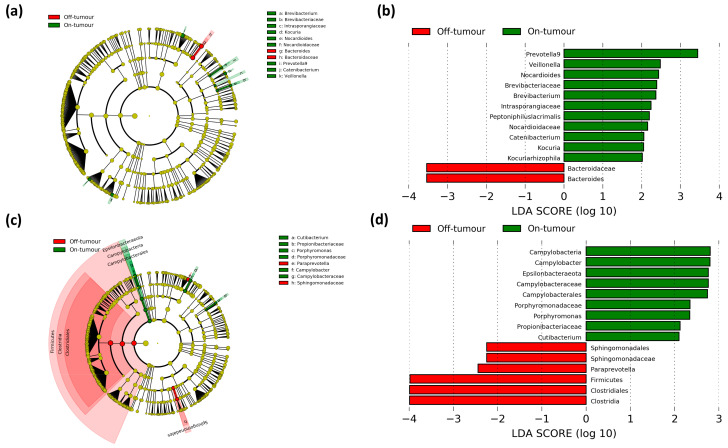
Paired comparison of on- and off-tumor taxa between treatment groups. LEfSe cladogram and histogram of LDA scores for differentially abundant bacterial taxa between on- and off-tumor microbiota in oral (**a**,**b**) and intravenous (**c**,**d**) iron-treated patients. Taxa and nodes highlighted in red were more significant in the off-tumor microbiota and green in the on-tumor microbiota. Taxa with an LDA score greater than 2 with a *p*-value ≤ 0.05 were considered statistically significant.

As the tumor-associated and tumor-adjacent microbiota showed differing outcomes following iron therapy in respect to bacterial taxa, we aimed to assess if the method of iron administration led to changes between paired on- and off-tumor microbiota (Figure 5a). Patients treated with oral iron showed their off-tumor microbiota being enriched with the *Bacteroidaceae* family and *Bacteroides* genus, while their on-tumor microbiota showed a higher abundance of *Nocardiaceae*, *Intrasporangiaceae*, and *Brevibacteriaceae* families and *Prevotella 9*, *Nocardioides*, *Kocuria*, *Brevibacterium*, *Veillonella*, and *Catenibacterium* genera (Figure 5c,d).

In contrast, patients treated with intravenous iron showed their off-tumor microbiota was enriched with the Firmicutes phylum and Clostridia class, along with a greater abundance of the Clostridiales and Sphingomonadales orders, the *Sphingomonadaceae* family, and the *Paraprevotella* genus. Whereas the on-tumor microbiota of patients treated with intravenous iron showed a higher abundance of the Epsilonbacteraeota phylum, Campylobacteria class, Campylobacterales order, *Campylobacteraceae, Propionibacteriaceae* and *Porphyromonadaceae* families, and *Campylobacter*, *Porphyromonas*, and *Cutibacterium* genera (Figure 5a,b). These results suggest that patients treated with oral iron had a more consistent tumor-associated and tumor-adjacent microbiota, showing only small changes at lower taxonomic levels, whereas patients treated with intravenous iron showed a much greater difference between their tumor-associated and tumor-adjacent microbiota, with major differences at the phylum, class, and order levels.

## 4. Discussion

A high incidence of colorectal cancer-associated iron deficiency leads to the requirement of therapeutic iron to correct anemia, with oral iron being the standard treatment due to low cost and convenience [21,22]. However, oral iron is associated with gastrointestinal side-effects and can increase colonic iron concentration, which has been shown to contribute to oncogenic signaling and colitis [23,24,25]. However, to our knowledge, this is the first study to investigate microbial outcomes of oral iron supplementation in anemic colorectal cancer patients.

This study demonstrates that the on- and off-tumor microbiota shows differential outcomes following oral and intravenous iron supplementation, demonstrating differences in α- and β-diversity, bacterial taxa, and predictive metagenomics. We believe the biological mechanisms underpinning these differences observed relate to the potential increase in colonic iron concentration following oral iron administration. This increased iron availability to colonic bacteria leads to competition for iron required for growth, with pathogenic bacteria having heightened iron acquisition mechanism and outcompeting probiotic bacteria [6]. The colonic iron concentration was not assessed within this study; however, previous studies have shown that oral iron administration leads to significantly greater fecal iron concentration compared to intravenous iron administration, supporting this proposed mechanism [26].

Off-tumor bacterial diversity is significantly different between oral and intravenous iron treatments, supporting a defined off-tumor microbial profile following each therapy. A previous study by Thomas et al. [27] showed that α-diversity is significantly increased in rectal cancer-associated microbiota compared to non-cancerous controls, as well as showing differentially β-diversity [27]. This suggests that an increased microbial diversity is potentially required to support cancer. Hence, the increase in off-tumor bacterial diversity in oral iron-treated patients (Figure 1) may be priming the colonic microbiota to support further colonic tumor development. In contrast, the on-tumor microbiota were more consistent between iron treatments, showing no difference in diversity metrics. This may potentially be due to pre-existing tumor microbial alterations, which may prevent oral iron from leading to major shifts in bacterial populations away from the cancer-defined microbiota.

On- and off-tumor bacterial genera that were more abundant in oral iron-treated patients include *Coprococcus, Prevotella*, and *Ruminococcus* (Figure 3a,b). A previous study by Flemer et al. [28] identified colorectal cancer-enriched microbiota, showing that *Coprococcus*, *Prevotella*, and *Ruminococcus* were all colorectal cancer-enriched genera [28]. Furthermore, a study by Kim et al. [29] found that the genera *Ruminococcus 2* and *Ruminococcaceae NK4A214* group, which were more abundant in oral iron-treated patients (Figure 3a,b), were enriched in colorectal cancer patient, whereas the genus *Lachnospiraceae NK4A136* group, which was more abundant in intravenous iron-treated patients (Figure 3a), was depleted in colorectal cancer patients [29]. This suggests that oral iron-treated patients have a greater abundance of colorectal cancer-enriched genera, whereas intravenous iron-treated patients have a greater abundance of colorectal cancer-depleted genera, implying oral iron may be utilized by the colonic microbiota promoting the expansion of cancer-associated bacteria that may support tumor development, while inhibiting potentially probiotic bacterial populations. However, what is not stated in these studies is whether these are driver or passenger bacteria in colorectal cancer.

Beneficial bacterial genera were differentially enriched between iron treatments, potentially including probiotic *Lactobacillus* which was more abundant in oral iron-treated patients (Figure 3a), whereas anti-inflammatory acetic and butyric acid-producing *Alloprevotella* and *Lachnospiraceae NK4A136* group were more abundant in intravenous iron-treated patients (Figure 3a,b) [30,31,32]. Collectively, the results of our study are consistent with previous findings by Lee et al. [26]. The authors showed that the fecal microbiota of oral and intravenous iron-treated inflammatory bowel disease patients had differential bacterial communities, supporting the results of our study, with the authors suggesting intravenous iron may be more beneficial for treating anemic Crohn’s disease patients, in order to limit microbial perturbations [26].

Predictive metagenomics shows the off-tumor microbiota of intravenous iron-treated patients has a greater abundance of bacterial enzymes involved in iron metabolism, compared to oral iron (Figure 4). Ferric-chelate reductase is a bacterial enzyme that catalases the reduction of siderophore-bound ferric iron to release ferrous iron [33]. Bacterial non-heme ferritin is a storage protein that binds ferrous iron, keeping it inert intracellularly [34]. Collectively, these can contribute to bacterial sequestration of colonic luminal iron. Hence, it is not biologically available to contribute to colonic inflammation and tumor initiation in the off-tumor colonic mucosa and potentially also sequesters iron away from the adjacent tumor microenvironment [35]. The off-tumor microbiota of intravenous iron-treated patients also showed an increased abundance of lactaldehyde dehydrogenase and 2-iminobutanoate/2-iminopropanoate deaminase. These bacterial enzymes both converged in pathways involved in lactate/pyruvate production and isoleucine biosynthesis. These enzymes and pathways are commonly associated with metabolite production in species of the *Lachnospiraceae* family, including the *Lachnospiraceae NK4A136* group which is more abundant in intravenous iron treated patients (Figure 3) [32,36]. Lactate and pyruvate are both anti-inflammatory metabolites that act to limit colonic inflammation [37,38,39]. Likewise, isoleucine has also been shown to inhibit intestinal inflammation, as well as acting to prevent colorectal cancer metastasis [40,41]. The on-tumor microbiota of intravenous iron-treated patients shows an increased abundance of cellulose synthase and N-sulfoglucosamine sulfohydrolase. Cellulose synthase is a bacterial enzyme that produces cellulose, which can inhibit colonic inflammation through modulating lipid metabolism and inactivating secondary bile acids [42,43]. Many bacteria of the Proteobacteria phylum are involved in bacterial biofilm cellulose production utilizing Cellulose synthase. Bacteria of the Proteobacteria phylum include the Alteromonadales order and *Enhydrobacter* genus which are both more abundant in the on-tumor microbiota of intravenous iron-treated patients (Figure 3) [44,45]. N-sulfoglucosamine sulfohydrolase produces glucosamine, which is anti-inflammatory and colorectal cancer-protective [46,47]. Collectively, this infers that the on- and off-tumor microbiota in intravenous iron-treated patients is associated with iron sequestration, along with anti-inflammatory and tumor protective metabolite production, compared to oral iron-treated patients. This implies that oral iron treatment potentially leads to a more procarcinogenic microbiota, due to the loss of protective probiotic bacterial populations that provide anti-oncogenic bacterial metabolites to the tumor microenvironment. However, it is important to note that it remains to be investigated whether the PICRUSt2 results are functional to further confirm the outcome of this study.

Comparison of paired on- and off-tumor microbiota shows differentially enriched bacterial taxa in the oral and intravenous iron-treated cohorts (Figure 5). The intravenous iron group shows substantial differences between the on- and off-tumor microbiota at the phylum level. In contrast, patients treated with oral iron show relatively few differences, primarily at the family and genus level. This suggests that oral iron treatment leads to a more consistent microbiota between cancerous and non-cancerous tissue. In contrast, intravenous iron-treated patients’ on- and off-tumor microbiota more relevantly reflect the more diverse bacterial populations typically found between tumor and non-tumor colonic tissue [28]. The clinical relevance of these differences in bacterial populations following each therapy has yet to be determined, but has the potential to be utilized as a future prognostic marker in the management of the disease [48].

This pilot study provided novel insights into microbial outcomes of iron therapy in colorectal cancer, suggesting oral iron may be a more deleterious microbial-altering iron therapy, whereas intravenous iron therapy may be more appropriate in this cohort of anemic patients. Despite the relatively small sample size of this study, significant differences were observed between iron treatment groups, which can form the foundation for further large-scale explorative studies. These studies could account for confounding variables, such as diet, which have the potential to lead to differential microbial populations, as well as including comparator colonic mucosal microbiota from healthy controls or pre-iron treatment. The relatively small window of therapeutic intervention in this study may show differential microbial outcomes compared to longer-term iron therapy. Nevertheless, this period relevantly reflects the common clinical intervention in anemic colorectal cancer patients prior to surgery. Furthermore, this study could be further validated through a more comprehensive study of bacterial metabolomics following iron therapy, as well as addressing the clinical relevance of differential microbial communities on long-term outcomes following surgery in these patients.

## 5. Conclusions

Collectively, this study suggested that the off-tumor microbiota is variable, depending upon iron supplementation. In contrast, the on-tumor microbiota seem to be more protected from the influence of iron supplementation, potentially due to pre-existing tumor bacterial dysbiosis. Oral iron-treated patients showed more consistent on- and off-tumor microbiota, whereas intravenous iron-treated patients showed large differences in bacterial populations between the on- and off-tumor microbiota. Predictive metagenomics from this study inferred that oral iron-treated patients have potentially more procarcinogenic on- and off-tumor microbiota compared to intravenous iron-treated patients. This implies intravenous iron may be a more beneficial to treat anemia in colorectal cancer patients, in order to prevent the microbial perturbations associated with oral iron supplementation. Future work should focus on determining the prognostic relevance of the differential gut bacterial populations following iron supplementation. This has the potential to allow the development of probiotic therapies to correct bacterial perturbations and potentially improve clinical outcomes for colorectal cancer patients following surgery.

## Figures and Tables

**Table 1:** Patient cohort demographics. Categorical variables are presented with percentages.

Patient Characteristics	Oral Iron (*n* = 16)	Intravenous Iron (*n* = 24)
Age	74.9 (7.4)	74.9 (9.5)
Male	8 (50%)	16 (67%)
Female	8 (50%)	8 (33%)
Height, m	1.66 (0.1)	1.70 (0.09)
Weight, kg	72.7 (17.4)	79.3 (17.3)
Inclusion Hb, g/L	100.3 (10.6)	98.8 (13.1)
Recruitment ferritin, μg/L *	24.5 (11.1–37.3)	23 (10–48.3)
Recruitment transferrin saturation, % *	2.9 (2.5–3.3)	2.8 (2.4–3.3)
Duration of iron treatment, days *	26.5 (15–43)	23.5 (15–40.5)
**Tumour Features**		
Tumour size, mm	48.5 (25.3)	42.4 (23.2)
* Tumour Stage*		
T≤2	1 (6.25%)	3 (12.5%)
T3	10 (62.5%)	18 (75%)
T4	5 (31.25%)	3 (12.5%)
* Tumour Location*		
Right colon	14 (87.5%)	17 (71%)
Left colon	2 (12.5%)	7 (29%)
**Preoperative Risk Assessment**		
* ASA fitness status classification*		
I–II	12 (75%)	10 (42%)
III–IV	4 (25%)	14 (58%)
CR-POSSUM mortality score, % *	3.6 (2.8–9.3)	3.5 (2.6–8.6)

Continuous variables are presented as mean value (standard deviation) or * median value (interquartile range). Hb, Hemoglobin. CR-POSSUM, ColoRectal Physiological and Operative Severity Score for the Enumeration of Mortality and Morbidity. ASA, American Society of Anesthesiologists.

## Data Availability

The datasets generated and analyzed during the study are available from the corresponding author on reasonable request.
